# Hierarchical additive effects on heterosis in rice (*Oryza sativa* L.)

**DOI:** 10.3389/fpls.2015.00738

**Published:** 2015-09-11

**Authors:** Zhiwu Dan, Jun Hu, Wei Zhou, Guoxin Yao, Renshan Zhu, Wenchao Huang, Yingguo Zhu

**Affiliations:** ^1^State Key Laboratory of Hybrid Rice, College of Life Sciences, Wuhan UniversityWuhan, China; ^2^Key Laboratory for Research and Utilization of Heterosis in Indica Rice, Ministry of Agriculture, Wuhan UniversityWuhan, China; ^3^The Yangzte River Valley Hybrid Rice Collaboration Innovation Center, Wuhan UniversityWuhan, China

**Keywords:** additive effects, heterosis, hierarchical structure, hybrids, multiplicative interactions, rice (*Oryza sativa* L.)

## Abstract

Exploitation of heterosis in crops has contributed greatly to improvement in global food and energy production. In spite of the pervasive importance of heterosis, a complete understanding of its mechanisms has remained elusive. In this study, a small test-crossed rice population was constructed to investigate the formation mechanism of heterosis for 13 traits. The results of the relative mid-parent heterosis and modes of inheritance of all investigated traits demonstrated that additive effects were the foundation of heterosis for complex traits in a hierarchical structure, and multiplicative interactions among the component traits were the framework of heterosis in complex traits. Furthermore, new balances between unit traits and related component traits provided hybrids with the opportunity to achieve an optimal degree of heterosis for complex traits. This study dissected heterosis of both reproductive and vegetative traits from the perspective of hierarchical structure for the first time. Additive multiplicative interactions of component traits were proven to be the origin of heterosis in complex traits. Meanwhile, more attention should be paid to component traits, rather than complex traits, in the process of revealing the mechanism of heterosis.

## Introduction

The world is currently facing the daunting task of addressing food and energy shortages. One cause of these shortages is the growing population. China needs to increase grain production by ∼25% to satisfy the huge national consumption by 2020 (Qiu, 2008; Wang et al., 2013). Another cause is the changing climate. Climate change could result in decreasing yields of staple food crops in most parts of the world from the 2030s onward (Challinor et al., 2014). Fortunately, the utilization of heterosis in crops has contributed greatly to global crop production improvement in recent decades (East, 1936; Whaley, 1944; Schnable and Springer, 2013). However, the mechanism of heterosis remains mysterious, and the profits that heterosis bring seem insufficient to meet worldwide demands. Discovering the mechanism of heterosis is an urgent task.

Heterosis describes improved performance of heterozygous F_1_ hybrids in terms of stature, biomass, size, yield, speed of development, fertility, resistance to diseases and insect pests, or to climatic rigors of any type compared to the average performance of their homozygous parental inbred lines (Birchler et al., 2006; Hochholdinger and Hoecker, 2007). Some terms have been proposed to describe heterosis. Additive effects describe phenotypes that are not significantly different from the average of the two parents (midparent value); partial dominance describes phenotypes that differ from the midparent but do not reach parental levels; dominance is not significantly different from the high or low parent value; and overdominance is substantially outside the range of the parental phenotypes, including above the high or below the low parent value (Hochholdinger and Hoecker, 2007; Schnable and Springer, 2013).

A wealth of studies on heterosis has been conducted since Darwin first scientifically recorded this phenomenon (Darwin, 1876). At the genomic level, overdominance was mainly found for grain yield (Yu et al., 1997; Lu et al., 2003; Larièpe et al., 2012), yield-related traits (Edwards et al., 1987; Stuber et al., 1987; Luo et al., 2001; Semel et al., 2006), plant height (Tang et al., 2007), and fiber quality (Wang et al., 2013). Epistasis and digenic interactions were also frequently detected (Li et al., 1997a, 2008; Yu et al., 1997; Hua et al., 2003; Shi et al., 2011; Guo et al., 2014). More importantly, additive effects appeared to be vital in the formation of heterosis as well (Edwards et al., 1987; Stuber et al., 1987; Li et al., 1997a; Luo et al., 2001, 2009; Hua et al., 2003; Shi et al., 2011; Qu et al., 2012). For traits especially resistance to disease, the genomic regions controlling *Phytophthora capsici* Leonian resistance could display additive effects, epistatic effects or both in pepper (*Capsicum annuum* L.; Thabuis et al., 2003). Furthermore, in the chickpea (*Cicer arietinum* L.), analysis for resistance to Ascochyta blight revealed that additive effects served a main role in Ascochyta blight resistant (Taleei et al., 2010).

At the transcriptomic level, genome-wide changes of gene expression have been documented in a variety of species and tissues. The majority of genes in hybrids showed additive expression in mouse liver (Cui et al., 2006), maize triploid endosperm (Guo et al., 2003), maize seedling (Stupar and Springer, 2006; Swanson-Wagner et al., 2006; Stupar et al., 2008), maize primary root (Hoecker et al., 2008), rice developing leaves and panicles (Wei et al., 2009), and *Arabidopsis* seedlings (Meyer et al., 2012). Furthermore, additively expressed genes were found to be positively associated with hybrid yield and heterosis (Guo et al., 2006; Jahnke et al., 2010; Thiemann et al., 2010, 2014). In addition, expression patterns of siRNA clusters in intrasubspecific rice hybrids mostly (78.8 and 77.7%) showed additive patterns in the two reciprocal hybrids (He et al., 2010). In addition to that, additive effects of two quantitative trait loci confer *Rhopalosiphum maidis* resistance in maize (Betsiashvili et al., 2015).

Consistent with gene-expression changes, in maize embryos and sunflower (*Helianthus annuus* L.) F_1_ hybrids, only small parts of the detected proteins exhibited non-additive accumulation (Marcon et al., 2010, 2013; Mohayeji et al., 2014). The results from a proteomic analysis of maize seeds showed that an additive pattern of protein abundances was established in heterotic hybrids and an additively balanced network but neither non-additive dominance nor overdominance regulates heterosis (Wang et al., 2014).

The reverse of heterosis is inbreeding depression (Semel et al., 2006; Chen, 2013). Additive epistatic loci were reported to have large effects on inbreeding depression in an intersubspecific rice F_4_ population (Li et al., 1997b). Overdominance resulting from epistatic loci was also found to be the primary genetic basis of inbreeding depression and heterosis in a rice population including 254 F_10_ recombinant inbred lines and two backcross hybrid populations (Luo et al., 2001; Mei et al., 2005). In a set of 148 F_9_ rice recombinant inbred lines, the epistatic effects of QTL pairs with additive and overdominant loci explained a larger portion of the total phenotypic variation for six yield-related traits (Luo et al., 2009).

In general, different experiments got mutually non-exclusive, different, or even conflicting results. Until now, no consensus has emerged, and single-locus approaches to studying heterosis are suggested to have limitations; therefore, progression to a quantitative genetic framework involving interactions in hierarchical networks may be fruitful (Birchler et al., 2010; Chen, 2013). Considering the truth that the phenotypes of heterosis are our best clues to its mechanism (Lippman and Zamir, 2006; Yao et al., 2013), we build a test-crossed rice population and analyzed the generation types of heterosis for 13 traits. We found, based on a hierarchical structure, that additive effects composed the fundamental level of heterosis for compound traits. Meanwhile, multiplicative interactions acted as the framework forming heterosis in complex traits.

## Materials and Methods

### Plant Materials

Eighteen inbred rice lines were used in this study (Supplementary Table S1), which were identified as indica or japonica with the InDel marker estimating method (Lu et al., 2009). R465 was provided by the China National Rice Research Institute. Mianhui725 was provided by Mianyang Academy of Agricultural Sciences. The line 9311K was provided by the Sichuan Academy of Agricultural Sciences. Qianlijing was provided by Sichuan Agricultural University. W1394, W1384, W1383, W1392, W1390, 110080 and Balilla were provided by Nanjing Agricultural University. The lines R4115, Liaoxing1 and Wuyunjing8 were provided by the Hunan Hybrid Rice Research Center. C418 was provided by Liaoning Academy of Agricultural Sciences. JR2 was provided by the Yunnan Academy of Agricultural Sciences. After heading, the panicles of the 18 inbred lines were bagged to insure the seed purity in July 2011 at the Engineering Research Center for Plant Biotechnology and Germplasm Utilization, Ministry of Education, Wuhan University, in Wuhan (N30° 32′ 22.44^′′^, E114° 22′ 18.21^′′^). Then, the seeds were sown in an experimental field at the Hybrid Rice Hainan Experimental Base of Wuhan University in Lingshui (N18° 30′ 22.12^′′^, E110° 2′ 10.72^′′^), Hainan Province, in December 2011. All 17 inbred lines were reciprocally crossed with Qianlijing from March to May 2012. A total of 34 bag hybrids were obtained through artificial emasculation hybridization.

### Trait Measurements

The seeds of 18 rice varieties and 34 hybrids were bagged in transparent plastic bags and submerged in water at 28°C for 48 h. Then, these seeds were transferred to an incubator at a constant temperature of 28°C for 24 h. Then, all seeds were planted in the experimental field of the Hybrid Rice Experimental Base of Wuhan University in Ezhou (N30° 22′ 19.82^′′^, E114° 44′ 59.17^′′^), Hubei Province, on 13 May 2012. The seedlings were transplanted to a farmland by adopting a randomized block design with three replications on 10 June 2012. A total of 10 individual plants of each replicate were planted at a spacing of 16.5 × 26.4 cm. To minimize the marginal effect, four Yuetai A plants, which are cytoplasmic male-sterile lines, were grown around these individuals. Five middle plants were used when phenotypic data were collected. The plant height was recorded four times. Height was recorded for the first time on 8 July 2012, and the data were defined as the SSPH. The second time was on 27 July 2012, and the data were defined as the ESPH. The third time depended on the heading dates, and the data were defined as the FSPH. Height was recorded for the fourth time when the seeds were mature, and the data were defined as the MSPH. The number of tillers with grains was considered the TPP. An average of 114 panicles of each hybrid combination was used to collect panicle traits, such as the PBN and the SBN. The YPP was weighed after drying, and the total grain number and EGN were determined using Seed Counting Machines (PME-1 Seed Auto-counting Machine, Shanke Equipment, Shanghai, China). The TGW was obtained by weighing one 1000 seeds. The total grain number per plant divided by the tiller per plant determined the GNP. The SSR was calculated by dividing the number of full grains per plant by the total grain number per plant. As a result of low germination and low survival ability, the amounts of some combinations were not sufficient for analysis; ultimately, 30 hybrid combinations remained. The means over replications were calculated for each trait and used in the data analyses.

### Data Analysis

The MPH was determined using the equation *MPH* = (*F_1_-MP)/MP*, where *F_1_* represents the value of a hybrid, and *MP* represents the mean of the parents. The trait data was analyzed using the software SPSS 19.0 (IBM SPSS Statistics for Windows, Version 19.0. Armonk, NY, USA). The degree of dominance was calculated with *d*/*a* = [hybrid-0.5(parent 1 + parent 2)]/abs (parent 1–parent 2) (Uzarowska et al., 2007) and aligned as in Stuber et al. (1987).

## Results

### Heterosis of the Rice Hybrids

**FIGURE 1:**
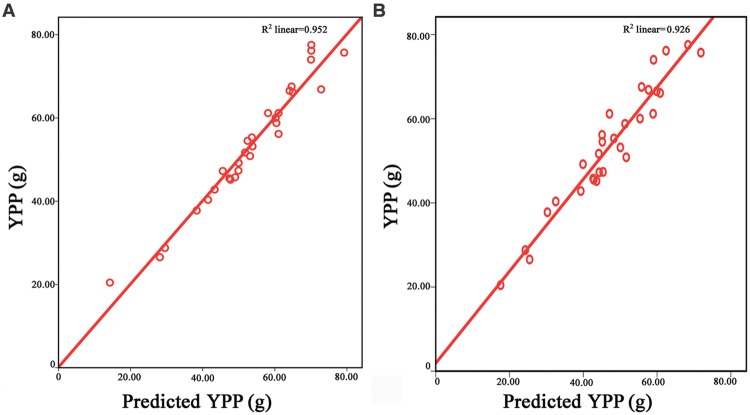
**Relationship between predicted YPP and observed yield values.** In **(A)**, the horizontal axis represents values predicted from the component traits of the hybrids. The equation used was YPP = 1.858^∗^TGW + 0.146^∗^GNP + 63.718^∗^SSR + 5.253^∗^TPP – 134.737. The vertical axis represents the true YPP of the hybrids. Red dots indicate the predicted YPP values. The red line is the interpolation line of the predicted YPP. In **(B)**, the horizontal axis represented the values predicted from the parental YPP. The ratios of F_1_/MP (F_1_ was trait value of the hybrid and MP was mean of its corresponding parents) for GNP, SSR, TPP, and TGW, respectively, were calculated first, and these ratios were multiplied by each other. Then, a value was obtained for each hybrid. Finally, this value was multiplied by the mean of YPP of the two parents to obtain a predicted YPP. The vertical axis represents the true YPP of the hybrids. Red dots predict the YPP from the parental lines. The red line is the interpolation line of the predicted YPP. The repeated times of the raw data for the five traits are 15. YPP, yield per plant; TGW, thousand-grain weight; GNP, grain number per panicle; SSR, seed-setting rate; TPP, tiller number per plant.

Grain number per panicle, SSR, TPP and TGW are component traits of rice yield (Xing and Zhang, 2010). To find out how these component traits contribute to YPP, YPP was designated the dependent variable and the above four component traits were designated as entered variables in a linear regression model with SPSS 19.0. The equation YPP = 1.858^∗^TGW + 0.146^∗^GNP + 63.718^∗^SSR + 5.253^∗^TPP – 134.737 was found to depict the relationship between component traits and YPP. Based on the equation, each hybrid received a predicted value for every trait, and the Pearson correlation coefficients between the predicted yield values and true yield data were as high as 0.976 (**Figure 1A**).

**FIGURE 2:**
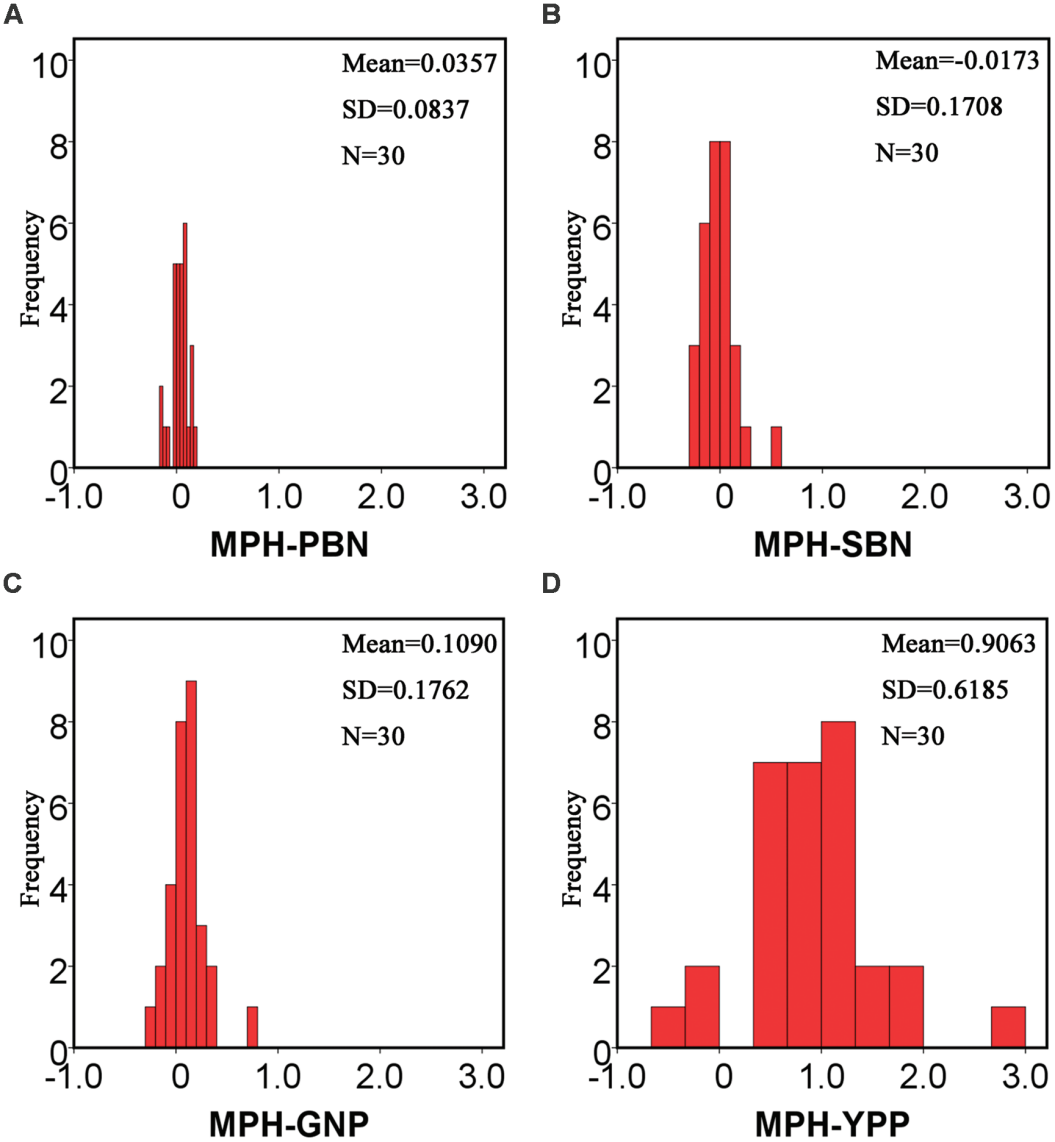
**Histograms of MPH for the PBN, SBN, GNP and yield per plant, respectively.** MPH for the PBN, SBN, GNP, and YPP of the hybrid population were calculated to study the changes in the degrees of heterosis for different traits. For MPH-PBN and MPH-SBN, the mean values were 0.0357 **(A)** and -0.0173 **(B)**, respectively. For MPH-GNP, the mean value was 0.109 **(C)**, which was much larger than the values of the primary and secondary branch number. For MPH-YPP **(D)**, the mean value was up to 0.9063. The low degree of heterosis in the component traits accumulated to form a high degree of heterosis in the complex traits. The repeated times of the raw data for PBN and SBN are about 114. N, number of the hybrids; SD, standard deviation.

Statistics of the MPH for all 13 traits in **Table 1** showed that, except for the SBN, all remaining traits manifested positive MPH. The highest average value of MPH was YPP, which was up to 0.906. Compared with YPP, the values of its component traits, such as TPP, GNP, SSR, and TGW, were smaller. Additionally, when we compared MPH-GNP and its component traits-primary branch number (MPH-PBN) and secondary branch number (MPH-SBN), the values for PBN and SBN were also smaller (**Figure 2**). The low degree of heterosis in the component traits combined to form a large magnitude of heterosis in complex traits.

**Table 1:** Relative mid-parent heterosis for all 13 traits.

Trait name	*N*	Average MPH^∗^	*SD*
GNP	30	0.109	0.176
YPP	30	0.906	0.619
TPP	30	0.265	0.378
SSR	30	0.058	0.113
TGW	30	0.131	0.068
PBN	30	0.036	0.084
SBN	30	-0.017	0.171
FGN	30	0.669	0.501
EGN	30	0.229	0.485
SSPH	30	0.116	0.106
ESPH	30	0.106	0.080
FSPH	30	0.164	0.106
MSPH	30	0.172	0.124

*Relative mid-parent heterosis for all thirteen traits (some traits in different stages) was calculated to investigate the performance of heterosis in different types of traits. The lowest average value of MPH was -0.017 for secondary branch number, and the highest was 0.906 for yield per plant. ^∗^ Average values of MPH for a trait. YPP, yield per plant; TGW, thousand-grain weight; GNP, grain number per panicle; SSR, seed-setting rate; TPP, tiller number per plant; SSPH, seedling stage plant height; ESPH, elongation stage plant height; FSPH, flowering stage plant height; MSPH, maturation stage plant height; PBN, primary branch number; SBN, secondary branch number; EGN, empty grain number per plant; FGN, full grains number per plant.*

To determine how the high magnitude of heterosis in YPP was produced, we multiplied the ratios of F_1_/MP (MP = the midparent value) for GNP, SSR, TPP and TGW, respectively, and received product R for each hybrid. Then, the mean measures of YPP for the two parents were used to multiply the corresponding R. Finally, a predicted YPP value was determined for each hybrid, and the correlation between the true field YPP data and the predicted data was calculated, where the *r* value was 0.962 (**Figure 1B**). Therefore, the heterosis of YPP was generated through multiplicative interactions of the component traits.

### Mode of Inheritance for All Traits

**FIGURE 3:**
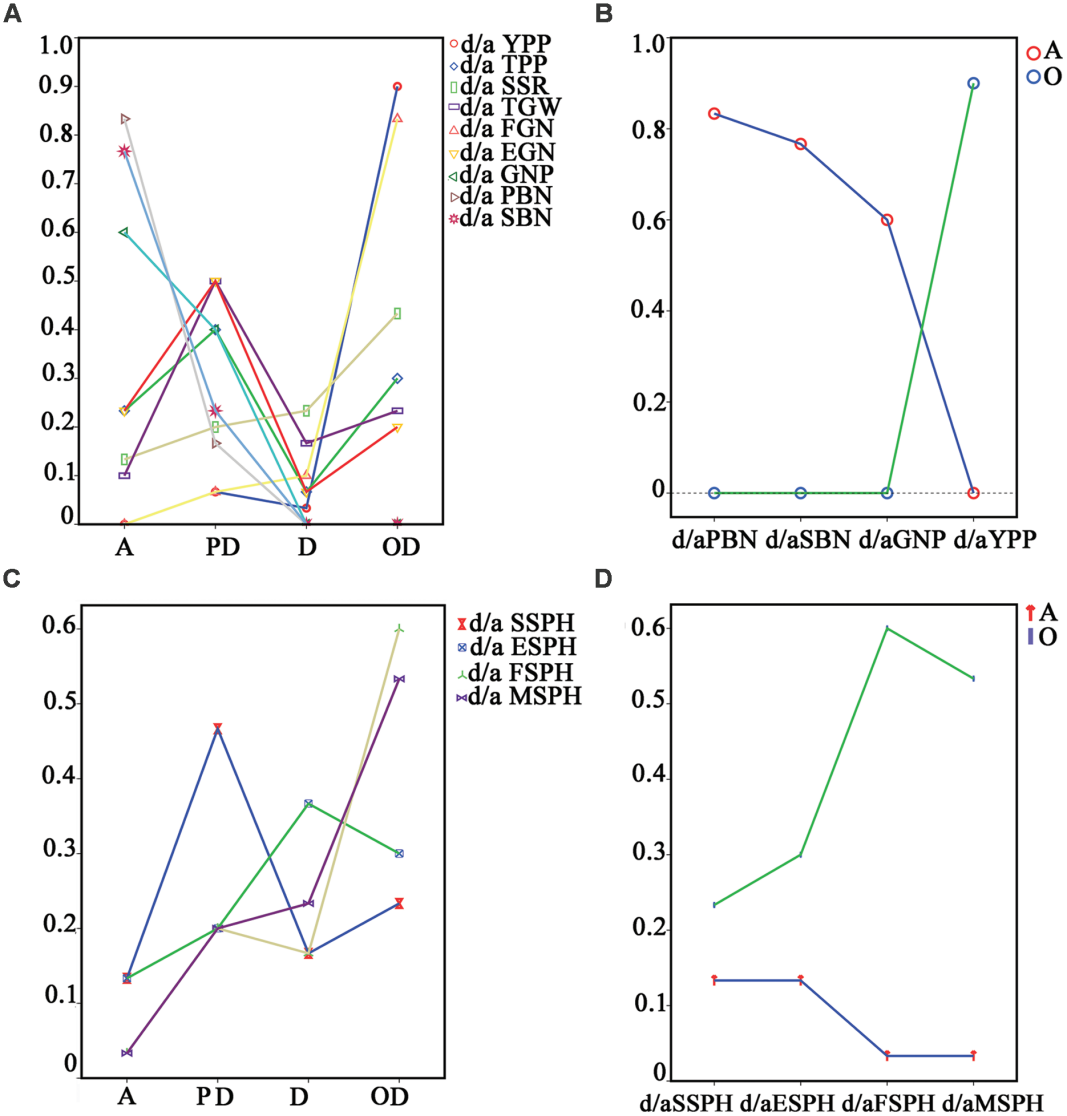
**Percentages of the different types of mode of inheritance for all 13 traits. (A)** and **(C)** were the percentages of different types of mode of inheritance for YPP and yield-related traits, and plant heights in four different growth stages of the testcrossing population, respectively. **(B)** described the percentage changes in PBN, SBN, GNP, and YPP for additive effects and overdominance, respectively. **(D)** was the percentage changes in plant height in seedling stage, elongation stage, flowering stage and maturation stage for additive effects and overdominance, respectively. No uniform trend in change of the percentages could be found when analysing the percentage changes from additive effects to overdominance in either the reproductive or vegetative traits **(A,C)**. The same trend in change in the percentage of additive effects decrease and overdominance increase were found in both the reproductive and vegetative traits **(B,D)**. The repeated times of raw data for EGN, FGN, SSPH, ESPH, FSPH, and MSPH are 15. A, additive effects; PD, partial dominance; D, dominance; OD, overdominance; YPP, yield per plant; TGW, thousand-grain weight; GNP, grain number per panicle; SSR, seed-setting rate; TPP, tiller number per plant; SSPH, seedling stage plant height; ESPH, elongation stage plant height; FSPH, flowering stage plant height; MSPH, maturation stage plant height; PBN, primary branch number; SBN, secondary branch number; EGN, empty grain number per plant; FGN, full grain number per plant.

Next, we analyzed the mode of inheritance for grain yield and yield-related traits. Additive effects, partial dominance, dominance and overdominance were found in the nine traits. To check whether one mode of inheritance is predominant or not, we calculated the percentages of the different types of mode of inheritance for the nine traits. Some traits (SSR, TGW, etc.) had all four types of modes. PBN and SBN demonstrated only additive effects and partial dominance. As shown in **Figure 3A**, an analysis of the percentage of changes from additive effects to overdominance, no uniform trend was observed in these traits. However, if we arranged the percentages from the component traits, PBN and SBN, to their corresponding compound trait, GNP, and further to GNP’s compound trait, YPP, two different changing trends were found: the percentage of additive genetic effect decreased, and the overdominance increased (**Figure 3B**). How about changes in the vegetative trait, plant height? In total, four growth stages of plant height were recorded in chronological order for analysis. Similar to reproductive traits, no uniform trend of percentage changes was found from additive to overdominance in the four stages (**Figure 3C**). However, to our surprise, two identical trends in changes of reproductive traits also existed for the additive effects and overdominance when the percentages were arranged in order of priority (**Figure 3D**).

### Hierarchical Additive Effects on Heterosis in Complex Traits

**FIGURE 4:**
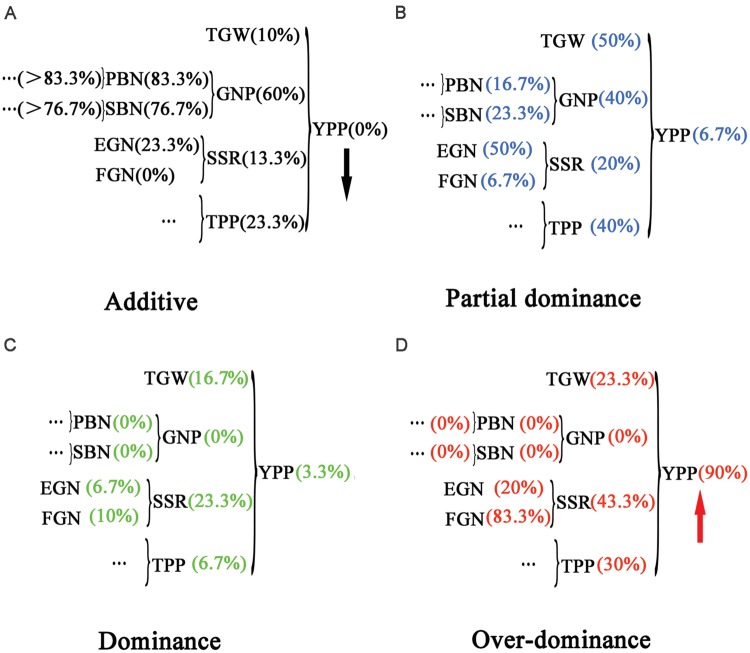
**Hierarchical distribution of yield and yield-related traits with percentages of different types of mode of inheritance.** A grain yield component trait map was drawn to describe the associations between yield and yield-related traits. Percentages of different types of mode of inheritance for the traits were marked in brackets. A decreasing trend was found for additive effects **(A)**, and an increasing trend was found for overdominance **(D)**, whereas no trend was found for partial dominance **(B)** and dominance **(C)**.

Next, the trends in **Figures 3B,D** inspired us to investigate the mode of inheritance for all the yield-related traits from the component to the complex traits. We developed the grain yield component trait-map shown in **Figure 4**. Yield-related traits were distributed in a hierarchical structure, and their corresponding percentage of additive effects, partial dominance, dominance, and overdominance were shown to the side, respectively. In **Figure 4A**, from left to right, the percentages of additive effects varied from high value to low value, displaying a decreasing trend. Furthermore, in **Figure 4D**, the percentages of overdominance had an increasing trend. Different from those changes observed in the additive effects and overdominance, no uniform trend could be found with percentages of partial dominance and dominance. Therefore, this outcome seems to suggest that additive effects and overdominance serve more important functions than dominance or partial dominance on hybrid traits. However, in PBN, SBN, and GNP, no overdominance could be detected (**Figure 4D**). Therefore, the remaining candidate was the additive effects.

Nonetheless, how additive effects act on heterosis are still unknown. In PBN and SBN, the values of MPH were 0.036 and -0.017 (**Table 1**; **Figure 2**), respectively, which suggested a low magnitude of heterosis. At the same time, the percentages of additive effects for PBN and SBN were as high as 83.3 and 76.7%, respectively. After integrating PBN and SBN into GNP, the value of MPH for GNP was 0.109, and the percentage of the additive effects was 60%. When TGW (0.131), GNP (0.109), SSR (0.058), and TPP (0.265) were combined to form YPP, the magnitude of MPH was as high as 0.906. Furthermore, the percentage of additive effects dropped to 0 simultaneously. Therefore, these results indicated that in low-level component traits, additive effects were prevalent and detectable. Moreover, the magnitude of heterosis was low. With the high-level complex traits, the additive effects were hidden and undetectable, and a high magnitude of heterosis emerged. Furthermore, the percentage of additive effects for component traits for traits such as PBN and SBN should be higher than 83.3 and 76.7%, respectively.

**FIGURE 5:**
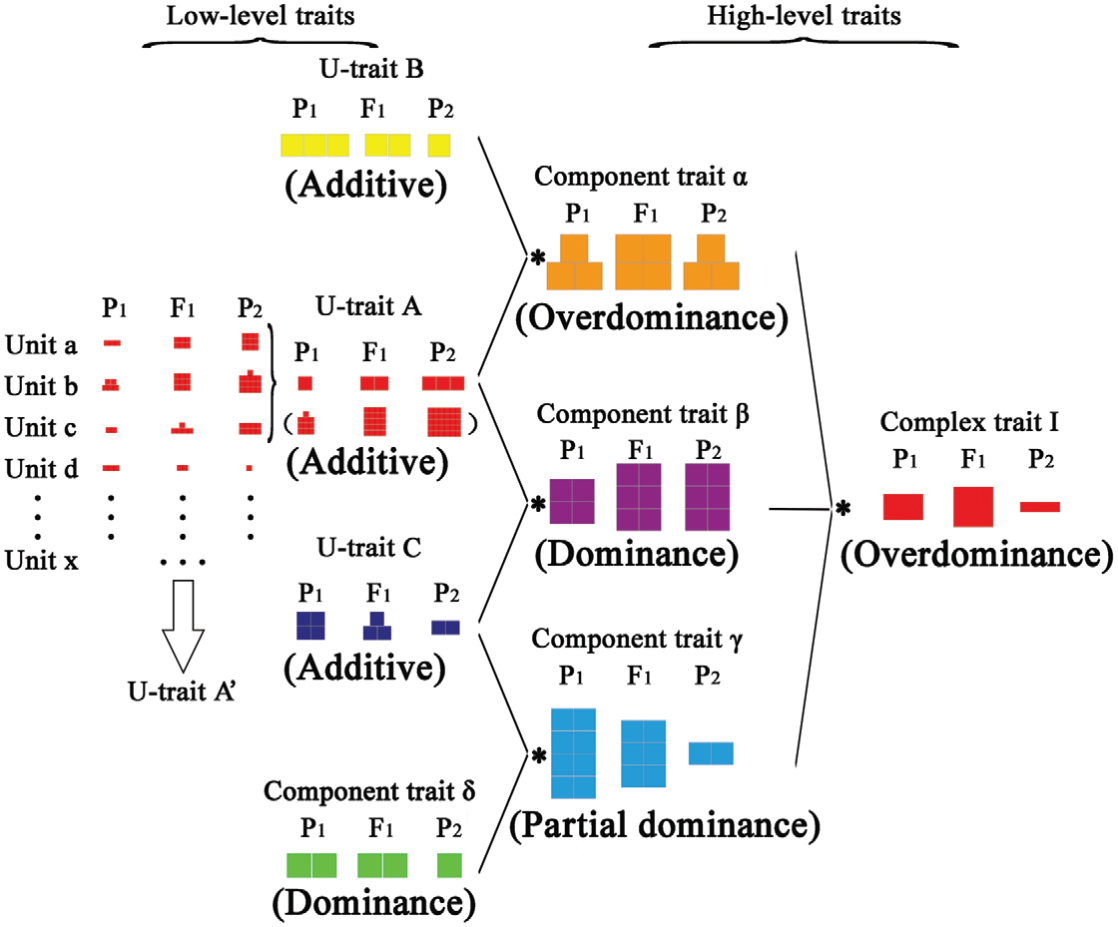
**Model of hierarchical additive effects on heterosis.** The model has three types of traits: unit-trait, component trait, and complex trait. The unit-trait has a series of units, which are additively presented. These units can form a unit-trait A′, whereas part of these units may form a unit-trait A, which becomes a component of another trait. Additive effects can be detected in u-traits, and they can join with each other to evolve into different component traits (component trait α, β, and γ) in a multiplicative way. The multiplicative combination of unit-traits is a crucial step in the performance of component traits, which depends on the relative amount of u-traits between parents. Then, various types of modes of inheritance can be detected in component traits. Meanwhile, a u-trait may combine a component trait (component trait δ) to form another component trait (component trait γ). Finally, component traits (α, β, and γ) can integrate into complex trait I. Different colors in the model represent different types of traits, and different size squares represent the varied trait values in hybrids and corresponding parents. Asterisks represent multiplicative interactions between the component traits.

By combining our finding of multiplicative interactions of component traits on heterosis for compound traits and the effects of additive on heterosis (**Figure 1B**, **Figures 2** and **4**), we present a model to elucidate hierarchical additive effects on heterosis (**Figure 5**). There are three types of traits: the unit-trait (u-trait), component trait, and complex trait. The u-trait consists of a series of unit elements that are additively presented. These elements may be gene-expression levels, protein contents, or a part of trait measures. Unit a, b, c, d, and so on can accumulate to form a u-trait A′, which also shows additive effects. Part of these units, unit a, b, and c, can form another u-trait A, which becomes part of a hierarchical structure. Parallel to u-trait A, u-trait B and u-trait C can join with u-trait A and evolve into component trait α and β, respectively, in a multiplicative way. This procedure is crucial, and the performance of hybrid component traits depends on the relative amount of u-traits between parents. U-trait A and u-trait B can manifest overdominance in the component trait α, whereas the component trait β displays dominance. Additionally, u-trait C can join with component trait δ, which shows dominance, and performs partial dominance effects for component trait γ. Eventually, the component traits α, β, and γ integrate into complex trait I with overdominance.

### Validation of the Model for Hierarchical Additive Effects on Heterosis

Next, we represent trait measures of the female parent and male parent as AAAA and BBBB, respectively. When the two parents were crossed to generate hybrids, based on our additive effects model, the reciprocal hybrids’ trait measures should be AABB and BBAA, respectively. Then, if we calculate the ratios of parent measures (AAAA:BBBB) and the reciprocal hybrids’ measures (AABB:BBAA), the ratios between the parents and hybrids should have no correlations. Whereas, after removing a quarter measures from the two hybrids, which is A and B, respectively, the ratios between the parents (AAAA:BBBB) and “reciprocal hybrids” (BAA:ABB) should have correlations. Furthermore, because the percentages of additive effects in grain yield component-trait map showed a decreasing trend, significant correlations could be detected in the low level component traits but not in the high level complex traits. To check whether this assumption was correct or not, we calculated the correlations between the ratios of AAAA:BBBB and BAA:ABB for all thirteen traits. Not surprisingly, we found significant correlations between the ratios for traits such as PBN, SBN and GNP, but no correlation for YPP (**Table 2**).

**Table 2:** Correlations between ratios of AAAA:BBBB, BAA:ABB and AABB:BBAA for all 13 traits.

Pearson correlation	BAA:ABB	AABB:BBAA
AAAA:BBBB-GNP	0.894†	-0.467‡
AAAA:BBBB-PBN	0.862†	-0.047‡
AAAA:BBBB-SBN	0.896†	-0.479‡
AAAA:BBBB-FGN	0.023‡	0.159‡
AAAA:BBBB-EGN	0.239‡	0.049‡
AAAA:BBBB-YPP	-0.002‡	0.172‡
AAAA:BBBB-TPP	0.390‡	-0.039‡
AAAA:BBBB-SSR	0.599^∗^	-0.453‡
AAAA:BBBB-TGW	0.889†	-0.678^∗^
AAAA:BBBB-SSPH	0.561^∗^	-0.033‡
AAAA:BBBB-ESPH	0.868†	-0.655^∗^
AAAA:BBBB-FSPH	0.593^∗^	-0.269‡
AAAA:BBBB-MSPH	0.630^∗^	-0.265‡

*AAAA and BBBB were trait values of the female and male parent, respectively. AABB and BBAA were trait values of the corresponding reciprocal hybrids. A quarter measure from the reciprocal hybrids, which was A and B, were removed from AABB and BBAA, respectively. Ratios between AAAA and BBBB, BAA and ABB, AABB and BBAA were calculated for all 13 traits. Correlations of these ratios were analyzed to validate the hierarchical additive model. ^∗^Correlation is significant at the 0.05 level (two-tailed). †Correlation is significant at the 0.01 level (two-tailed). ‡No significant correlation.*

## Discussion

The rice cultivar Qianlijing was chosen as the tester. It has ∼400 grains per panicle, which is the highest in the 18 inbred rice lines. The PBN and SBN are also the largest, which is ∼2–7 times as high as other inbred lines. Meanwhile, Qianlijing has the least amount of tillers, relative lower TGW and shorter plant height. These phenotypes are advantageous for us to distinguish the four types of mode of inheritance.

### Additive Effects on Heterosis

East (1936) suggested that *“The cumulative action of the non-defective allelomorphs of a given gene approaches the strictly additive as they diverge from each other in function”* when investigating heterosis. Today, this viewpoint is surprisingly accurate. In maize triploid endosperm, most gene-expression patterns were not distinguishable from additivity and the expression level of additive gene action was proportional to the parental contribution [(2^∗^*P_M_* + *P_P_*)/3, where *P_M_* is the expression level of the maternal inbred line and *P_P_* is the expression level of the paternal inbred line; Jahnke et al., 2010]. In reciprocal triploid maize hybrids, trait measures of eight of nine traits showed no significant difference in their respective weighted midparent means (the midparent means were calculated as two-thirds of one parent and one-third of the corresponding parent; Yao et al., 2013). *Cis*-regulation seems to have profound influences on additive expression patterns in the F_1_ hybrids (Doss et al., 2005; Stupar and Springer, 2006).

In this study, additive effects were detected in various traits with different percentages. For traits such as PBN and SBN, secondary branches are differentiated by second-order lateral meristems, which are produced by the primary branches (Xing and Zhang, 2010). For EGN and FGN, empty grains need more procedures to produce full grains, such as double fertilization, embryogenesis, and grain filling. Additionally, for plant heights at different stages of development, later growth stages have more structural components, such as more internodes, involved to form the whole trait. The above three types of traits had a similar decreasing trend of additive effects percentages. Hence, for same type traits or same traits in different growth stages, traits with fewer procedures had more additive effects. Furthermore, traits with more procedures or parts could reduce the percentage of additive effects. This type of “reduction” might be interpreted as “use,” which means secondary branches could use the additive portion in the primary branches.

### Hierarchical Structure of the Complex Traits

Yield is a compound character that is a product of the expression and complex interactions of the component characteristics (Williams, 1959; Sage and Hobson, 1973). One study in the tomato demonstrated that even single-gene yield heterosis is also based on multiplicative interactions between component traits (Krieger et al., 2010). Whether a trait is identified as component or compound is relative to its corresponding analyzed traits. A trait (GNP) might be a component trait of another complex trait (YPP) and a combination outcome of several u-traits (PBN and SBN) simultaneously. Therefore, hierarchical structures could be found in heterosis for complex traits.

The multiplicative effects of components on heterosis are widely known in complex characteristics (Schnel and Cockerham, 1992; Melchinger et al., 1994). In the same group of units (unit a, b, c, d, etc.) in **Figure 5**, simple accumulative additive effects formed unit-traits. Furthermore, for different types of unit-traits and component traits, multiplicative additive effects functioned on a high level. The interaction results of these components depended on the relative amount of matched components, which could be additive, partial dominance, dominance, or overdominance.

### Optimal Balances between Component Traits Stimulated Heterosis in Complex Traits

Components traits are not completely independent of each other, and a compensatory nature exists in these traits (Sage and Hobson, 1973). Negative correlation between number and weight of grain in rice (Li et al., 1997a), whole-plant phenotype associations in tomato (Schauer et al., 2006), and correlations for yield-correlated traits in rapeseed (Shi et al., 2011) are the consequences of the tight connections between component traits. This may indicate feedback regulation of biological networks in complex traits of heterosis provided by metabolites (Chen, 2013). Because of the compensatory nature in component traits, a high magnitude of yield heterosis cannot be achieved by greatly increasing part of the component traits (Sage and Hobson, 1973). When a component is pushed too far from its normal level, physiological breakdown occurs (Williams, 1959), which could be a higher degree of heterosis for biomass and increased reproductive isolation in intersubspecific rice hybrids (Dan et al., 2014). Furthermore, in breeding programs, a key factor for breeders is to understand the correlations between traits and the extent to which they can be uncoupled (Robson et al., 2013).

In combination, the fact that not every hybrid manifests heterosis in a population and that interactions between components are quite crucial to the performance of hybrids, heterosis likely necessitates optimal balances in component traits. In hybrids, the compensation between component trait functions identically to that of their parents. However, the hybrids obtain the opportunity adjust the amount of compensation in the component traits (2 multiplied by 2 to get 4 in hybrids, rather than 1 (or 3) multiplied by 3 (or 1) to get 3 in the parental lines). One example is the additive by additive interaction between the *B* and *Pl* gene that can result in the overdominant expression of phenotypes *A1, A2*, and *Bz1* for red pigmentation rather than green in maize (Dooner and Robbins, 1991; Springer and Stupar, 2007). However, this type of balance might be difficult to be implemented on account of the network comprising various related traits. Breakthroughs might be made by analyzing unit traits because they have relatively easier genetic patterns. Furthermore, the most important thing should be to define the proportional role of single-trait heterosis from pleiotropic heterosis (Kaeppler, 2011), namely determining the component trait contribution to complex traits of heterosis (Robson et al., 2013). Classification and subdivision to the present traits and new estimating methods for new traits might be needed.

## Conclusion

A hybrid rice population was constructed to investigate the formation mechanism of heterosis in 13 traits. The results demonstrated that, based on the hierarchical structure of complex traits, additive effects from component traits generated heterosis through multiplicative interactions in rice hybrids. Furthermore, new breakthroughs might be made by investigating the mechanism of heterosis in basal component traits.

## Author Contributions

ZD, JH, WH, and YZ designed the research; ZD, WZ, GY, and RZ performed research; ZD and WH analyzed data and wrote the manuscript. All authors read and approved the manuscript.

## Conflict of Interest Statement

The authors declare that the research was conducted in the absence of any commercial or financial relationships that could be construed as a potential conflict of interest.

## Abbreviations

EGN: empty grain number per plant
ESPH: elongation stage plant height
FGN: full grains number per plant
FSPH: flowering stage plant height
GNP: grain number per panicle
MPH: relative mid-parent heterosis
MSPH: maturation stage plant height
PBN: primary branch number
SBN: secondary branch number
SSPH: seedling stage plant height
SSR: seed-setting rate
TPP: tiller number per plant
TGW: thousand-grain weight
YPP: grain yield per plant
